# Long-Term Outcomes Associated With Posterior Fossa Syndrome in Survivors of Childhood Medulloblastoma

**DOI:** 10.1001/jamanetworkopen.2025.59376

**Published:** 2026-02-19

**Authors:** Supriya Sarvode, Rikeenkumar Dhaduk, Yan Chen, Siddhant Taneja, Johnnie K. Bass, Robyn Partin, Kristin Szymanek, Matthew Wogksch, Heather M. Conklin, Darcy Raches, Thomas E. Merchant, Paul Klimo, Amar Gajjar, Kevin R. Krull, Raja B. Khan, Gregory T. Armstrong, Kirsten K. Ness, Giles W. Robinson, Sedigheh Mirzaei, Tara M. Brinkman, Melissa M. Hudson, Nicholas S. Phillips

**Affiliations:** 1 Department of Oncology, St Jude Children’s Research Hospital, Memphis, Tennessee; 2 Department of Epidemiology and Cancer Control, St Jude Children’s Research Hospital, Memphis, Tennessee; 3 Department of Rehabilitation Services, St Jude Children’s Research Hospital, Memphis, Tennessee; 4 Department of Hematology, St Jude Children’s Research Hospital, Memphis, Tennessee; 5 Department of Psychology and Biobehavioral Sciences, St Jude Children’s Research Hospital, Memphis, Tennessee; 6 Department of Radiation Oncology, St Jude Children’s Research Hospital, Memphis, Tennessee; 7 Department of Surgery, St Jude Children’s Research Hospital, Memphis, Tennessee; 8 Department of Pediatric Medicine, St Jude Children’s Research Hospital, Memphis, Tennessee; 9 Department of Biostatistics, St Jude Children’s Research Hospital, Memphis, Tennessee

## Abstract

**Importance:**

Posterior fossa syndrome is a debilitating surgical complication affecting communication, motor skills, mood, language, and working memory in children treated for posterior fossa tumors. Although many recover from acute symptoms, the lifetime impact of posterior fossa syndrome remains unknown.

**Objective:**

To evaluate the long-term neurological, neurocognitive, social, and quality of life outcomes associated with posterior fossa syndrome among survivors of medulloblastoma.

**Design, Setting, and Participants:**

This retrospective cohort study included survivors of childhood medulloblastoma diagnosed between 1985 and 2012, with more than 5 years from diagnosis. All participants were treated at a tertiary academic center. Data were analyzed from January 1, 2024, to December 1, 2025.

**Exposure:**

History of posterior fossa syndrome.

**Main Outcomes and Measures:**

Outcomes of interest included neurocognitive functioning (attention, cognitive flexibility, and visuomotor speed), neurological outcomes (cerebellar dysfunction, cranial nerve disorders, dysarthria, headaches, movement disorders, paralytic disorders, peripheral motor or sensory neuropathy, and seizures), physical performance, and social functioning. Statistical analysis included Mann-Whitney *U*, χ^2^, or Fisher exact tests, with multivariable linear regression used to assess the associations of posterior fossa syndrome with outcomes while adjusting for confounders.

**Results:**

A total of 158 participants (median [range] age at assessment, 25 [11–44] years; 96 [60.8%] male) were assessed, including 37 (23%) who developed posterior fossa syndrome and 121 controls who did not, with no differences in age at diagnosis, radiation dose, or age at assessment a median (range) follow-up of 14.2 (7.8-33.1) years. In adjusted models, participants with posterior fossa syndrome performed worse than those without in focused attention (β = −1.04 [95% CI, −1.62 to −0.45]; *P* < .001), motor-processing speed (β = −0.62 [95% CI, −1.16 to −0.09]; *P* = .02), cognitive flexibility (β = −0.85 [95% CI, −1.44 to −0.27]; *P* = .005), visuomotor processing speed (β = −0.65 [95% CI, −0.97 to −0.33]; *P* < .001), and physical performance test scores (β = −3.65 [95% CI, −5.36 to −1.93]; *P* < .001). Participants with posterior fossa syndrome were also more likely to require assistance with routine daily needs (odds ratio, 8.00 [95% CI, 2.56 to 25.04]; *P* < .001).

**Conclusions and Relevance:**

In this long-term cohort study of survivors of medulloblastoma, individuals with history of posterior fossa syndrome exhibited persistent neurocognitive and physical deficits compared with those without history of posterior fossa syndrome. Despite resolution of acute postoperative symptoms, posterior fossa syndrome was associated with lasting impairment, underscoring the need for improved surgical approaches, continued surveillance, and tailored interventions to optimize functional outcomes.

## Key Points

**Question:**

What are the long-term outcomes associated with posterior fossa syndrome in survivors of childhood medulloblastoma?

**Findings:**

In this cohort study of 158 survivors of medulloblastoma 5 or more years from diagnosis, 23% developed posterior fossa syndrome. Compared with participants without posterior fossa syndrome, those with posterior fossa syndrome had significant deficits in attention, processing speed, and cognitive flexibility, and physical function and were more likely to require assistance with routine needs.

**Meaning:**

This cohort study found that posterior fossa syndrome was associated with lasting neurocognitive and physical deficits in survivors of medulloblastoma, emphasizing the need for strategies to minimize surgical morbidity, continued interventions, and support in this high-risk population to improve outcomes among survivors.

## Teaser

This cohort study evaluates the long-term neurological, neurocognitive, social, and quality of life outcomes associated with posterior fossa syndrome among survivors of childhood medulloblastoma.

## Introduction

Posterior fossa syndrome, also known as *cerebellar mutism syndrome*, is a severe and debilitating postoperative complication after posterior fossa tumor resection,^1,2^ with reported incidence ranging from 6% to 39% because of variability in diagnostic criteria, timing of assessment, and treatment era.^3,4,5,6,7^ Symptoms typically emerge 1 to 2 days after surgery and include transient mutism or reduced speech, ataxia, hypotonia, motor weakness, and emotional lability.^2^ More recent, methodologically rigorous cohorts using standardized definitions report a more consistent incidence of approximately 20% to 30%.^4,8,9^

Posterior fossa syndrome has been described under multiple labels over the past 35 years, including *cerebellar speech syndrome*, *pseudobulbar palsy*, *mutism without behavioral impairment*, and *cerebellar cognitive affective syndrome*.^10,11,12,13,14,15^ To encompass the full spectrum of neuropsychiatric and behavioral manifestations, we use the term *posterior fossa syndrome* throughout.^16^ Recovery is often prolonged and incomplete, with persistent cognitive, affective, and motor sequelae^17^ that place significant burdens on families and the health care system.^18^

Clinical and anatomic risk factors for posterior fossa syndrome include tumor histology, size, location, invasiveness, patient age, and the medulloblastoma molecular subgroup.^4,19^ Neuroimaging suggests that injury to the proximal dentatothalamocortical tract and disruption of the fastigial nucleus and/or superior cerebellar peduncles may play a critical role in posterior fossa syndrome pathophysiology.^20,21^ The severity of mutism, dysmetria, and ataxia is associated with dentatothalamocortical tract injury, and functional magnetic resonance imaging demonstrates cerebrocerebellar abnormalities extending beyond the surgical field.^22^

Survivors of medulloblastoma face long-term morbidity,^23,24^ particularly in processing speed, task efficiency, and working memory.^25,26^ Survivors of medulloblastoma with posterior fossa syndrome show greater early declines,^27^ raising concerns about accelerated cognitive aging and early dementia. However, long-term neurobehavioral outcomes in adolescents and young adults with posterior fossa syndrome remain poorly characterized. Rigorous evaluation of chronic morbidity is essential to guide clinical care and targeted interventions.

The St. Jude Lifetime Cohort Study (SJLIFE) provides a unique opportunity to assess neurologic, neurocognitive, social, and quality of life outcomes among long-term survivors of medulloblastoma and to examine differences by posterior fossa syndrome status. We investigated these outcomes in a prospectively followed cohort, hypothesizing that survivors with posterior fossa syndrome would show worse neurologic, intellectual, and academic performance than those without posterior fossa syndrome.

## Methods

This cohort study was conducted as part of SJLIFE, retrospective cohort with prospective follow-up of survivors of childhood cancer with at least 5 years since diagnosis, diagnosed between 1962 and 2012 and treated at St. Jude Children’s Research Hospital.^28^ SJLIFE was approved by the St. Jude Children’s Research Hospital institutional review board, and all participants provided written informed consent. This study is reported following the Strengthening the Reporting of Observational Studies in Epidemiology (STROBE) reporting guideline

### Study Design and Population

The St. Jude Children’s Research Hospital brain tumor program began in 1985; thus, all survivors of medulloblastoma in this analysis were diagnosed between 1985 and 2012. Long-term outcome assessment of survivors began in 2007. Participants undergo comprehensive clinical evaluation and neurocognitive testing every 5 years,^29^ with additional assessments, including physical function testing (eMethods in Supplement 1) and questionnaires capturing sociodemographic characteristics, health status, and medical history. Race and ethnicity were categorized as White, non-Hispanic and other. Further categorization of the other race group and ethnicity is not feasible owing to small cell sizes. This category includes Asian; Black; Caribbean; Mexican or Chicano; not otherwise specified Spanish, Hispanic, or Latino; Puerto Rican; and South or Central American. The SJLIFE cohort has been previously described.^28,30^

Eligibility criteria included a diagnosis of medulloblastoma at age 3 years or older and completion of at least 1 on-campus SJLIFE evaluation. Children younger than 3 years at diagnosis were excluded because they typically receive infant treatment protocols involving high-dose chemotherapy (with or without autologous stem cell transplant) and radiation-delaying strategies. Posterior fossa syndrome classification was based on physician documentation in the medical record. It required mutism or markedly reduced speech output (eg, limited to 2- to 3-word phrases), with or without accompanying behavioral dysregulation, ataxia, or hypotonia. For survivors diagnosed before standardized posterior fossa syndrome definitions were widely adopted, classification relied on explicit clinical documentation. Survivors with unrelated neurologic or neurogenetic disorders were excluded. Among 337 eligible survivors with at least 5 years from diagnosis, 231 were aged 3 years or older at diagnosis, and 158 (37 with posterior fossa syndrome; 121 without posterior fossa syndrome) completed a campus evaluation before the analysis cutoff (April 30, 2020) (eFigure in Supplement 1).

Because younger age at craniospinal irradiation and at diagnosis are strongly correlated and both are associated with posterior fossa syndrome risk,^31,32,33^ only age at diagnosis was included in multivariate models. Treatment details, including surgery, radiation, and chemotherapy, were abstracted from medical records. Patients with medulloblastoma were stratified as average risk or high risk based on modified Chang tumor staging, patient age, and extent of surgical resection and received risk-adapted craniospinal photon irradiation followed by chemotherapy..

### Neurocognitive Assessment

Participants completed a standardized 2-hour evaluation administered by a trained examiner. The battery assessed intelligence, attention, academics, processing speed, memory, and executive function and was repeated every 5 years.^18,19,20^ Scores were converted to age-adjusted *z* scores normalized to the general population (mean [SD], 0 [1]); impairment was defined as lower than −1.28 (10th percentile). Participants also completed the 32-item Neurocognitive Questionnaire^34^ on a validated scale (range, 1-3; higher scores indicate more problems).

### Physical Performance and Neurologic Assessment

A clinical exercise physiologist conducted physical performance testing, including resting vitals, the 7-item Physical Performance Test,^35^ and timed up-and-go evaluation.^36^ Additional performance assessments are detailed in the eMethods in Supplement 1.^35,36,37,38,39,40,41,42,43^^,^ Neurologic symptoms, including dysarthria, were obtained through medical record review, self-report, or physical examination and graded using the modified National Cancer Institute Common Terminology Criteria for Adverse Events (version 4.03).^44^ Grade 2 or greater was considered impaired. Motor function was assessed through neurologic examination as part of the standardized SJLIFE evaluation.

### Hearing Assessment

Audiologic evaluations measured pure tone thresholds (0.25-8.00 kHz). Hearing loss was classified using the International Society of Pediatric Oncology ototoxicity grading scale, with grade 3 or higher considered severe.^45,46,47^ We used grades 3 to 4 to define severe hearing loss because these levels most consistently correspond to clinical recommendations for hearing aids or a cochlear implant at the end of therapy. For asymmetrical hearing loss, the least-impaired ear was used to determine severity.

### Social and Quality of Life Assessment

Quality of life was evaluated using the Short Form-36 (SF-36),^48^ with impairment defined as a *T*-score less than 40, reflecting 1 SD below age-adjusted norms (population mean [SD], 50 [10]). Questionnaires assessed marital status, employment, education, and daily functioning.

### Statistical Analysis

Distributions of continuous variables were visually inspected to assess normality, and potential outliers or influential observations were evaluated. Baseline demographic and treatment characteristics were compared between participants with and without posterior fossa syndrome using the Mann-Whitney *U* test for continuous variables and the χ^2^ test or Fisher exact test for categorical variables, as appropriate.

Associations between posterior fossa syndrome and outcomes were examined using multivariable linear regression for continuous outcomes and multivariable logistic regression for binary outcomes. Models adjusted for age at diagnosis, years since diagnosis, sex, extent of surgical resection, craniospinal irradiation dose, cerebrospinal fluid (CSF) shunt placement, and treatment protocol; models of neurocognitive outcomes additionally adjusted for severe hearing loss. For continuous outcomes, age-adjusted *z* scores from the SJLIFE registry were used, and coefficients represent adjusted mean differences in *z* score units. No baseline covariates had missing data. Participants with missing outcome data were excluded from the respective analyses, and models were fit using all available observations. Because the mechanism of missingness cannot be fully assessed, results should be interpreted accordingly. Cumulative incidence was compared using the log-rank test. *P* values were 2-sided, and statistical significance was set at *P* ≤ .05. All analyses were performed using SAS software version 9.4 (SAS Institute). Data were analyzed from January 1, 2024, to December 1, 2025.

## Results

A total of 158 patients (median [range] age at assessment, 25 [11-44] years; 96 [60.8%] male) were assessed, including 37 (23%) who developed posterior fossa syndrome and 121 (76.6%) who did not. Patients with vs without posterior fossa syndrome did not differ in age at diagnosis, radiation dose, or age at assessment (Table 1). Neurological, neurocognitive, physical performance, and social outcomes were assessed at a median (range) of 14.2 (7.8-33.1) years after diagnosis of medulloblastoma. Participants with posterior fossa syndrome were more likely to have a CSF shunt (22 participants [59.5%] vs 36 participants [29.8%]; *P* = .001), and a higher proportion underwent multiple surgical resections (15 participants [40.5%] vs 30 participants [24.8%]), although the difference did not reach statistical significance (*P* = .06). Participants with posterior fossa syndrome were more likely to have a CSF shunt (22 participants [59.5%] vs 36 participants [29.8%]; *P* = .001) and less likely to have a body mass index (calculated as weight in kilograms divided by height in meters squared) greater than 25 (13 participants [35.1%] vs 68 participants [56.2%]; *P* = .02), while all other baseline characteristics were similar between groups (Table 1). Additionally, there were no baseline differences between individuals who participated and those who did not (eTable 1 in Supplement 1), indicating the representativeness of the study sample. There was no significant difference in age at the time of diagnosis between participants with posterior fossa syndrome (median [range] age, 7.5 [3.2-17.4] years) and those without posterior fossa syndrome (median [range] age, 9.1 [3.0-22.3] years). Age at diagnosis and age at craniospinal irradiation were highly correlated clinically; all comparisons were adjusted using age at diagnosis.

**Table 1.** Demographic and Treatment Characteristics of Survivors of Medulloblastoma With and Without Posterior Fossa Syndrome

Characteristic	Participants, No. (%)		*P* value
	Posterior fossa syndrome (n = 37)	No posterior fossa syndrome (n = 121)	
Sex			
Female	12 (32.4)	50 (41.3)	.33^a^
Male	25 (67.6)	71 (58.7)	
Age, median (range), y			
At diagnosis	7.5 (3.2-17.4)	9.1 (3.0-22.3)	.13^b^
At assessment	24.5 (14.6-43.2)	25.0 (10.8-44.3)	.96^b^
Time from diagnosis, median (range), y	18.4 (9.1-31.9)	14.2 (7.8-33.1)	.17^b^
Craniospinal radiation, Gy			
<30	19(51.4)	65(53.7)	.80^a^
≥30	18(48.6)	56(46.3)	
Race and ethnicity			
Other^c^	8 (21.6)	35 (28.9)	.38^a^
White non-Hispanic	29 (78.4)	86 (71.1)	
Surgical resections, No.			
1	22 (59.5)	91 (75.2)	.06^a^
>1	15 (40.5)	30 (24.8)	
Extent of resection			
Gross total resection	25 (67.6)	96 (79.3)	.14^a^
Subtotal resection	16 (43.2)	35 (28.9)	.10^a^
Biopsy only	1 (2.7)	5 (4.13)	.38^d^
Shunt	22 (59.5)	36 (29.8)	.001^a^
BMI >25	13 (35.1)	68 (56.2)	.02^a^

Abbreviation: BMI, body mass index (calculated as weight in kilograms divided by height in meters squared).

^a^ Calculated using the χ^2^ test.

^b^ Calculated using Fisher exact test.

^c^ Further categorization of the other race group and ethnicity is not feasible owing to small cell sizes. This category includes Asian; Black; Caribbean; Mexican or Chicano; not otherwise specified Spanish, Hispanic, or Latino; Puerto Rican; and South or Central American.

^d^ Calculated using the Mann-Whitney *U* test for continuous variables.

### Long-Term Neurological Impairment

The prevalence of cerebellar dysfunction, cranial nerve disorders, and peripheral sensory and motor neuropathy increased with increasing time from diagnosis in both groups with and without posterior fossa syndrome (Figure). Although no significant differences between groups were observed in time-to-event analyses, the cumulative incidence difference of cranial nerve disorders 20 years after diagnosis was 33% (95% CI, 0% to 68.7%), and the difference for cerebellar dysfunction was 12% (95% CI, 0% to 39.0%) (Figure). Among participants with posterior fossa syndrome, the median (IQR) time to speech follow-up was 2.11 (1.05-3.37) months (range, 0.03-19.47 months). Of 10 neurological outcomes examined, only severe hearing loss differed between groups in unadjusted analyses, although this association was not significant after adjustment (odds ratio [OR], 1.84 [95% CI, 0.58-5.82]; *P* = .30) (eTable 2 and eTable 3 in Supplement 1). Sensory Organization Test performance did not differ significantly between participants with and without posterior fossa syndrome.

**Figure.**
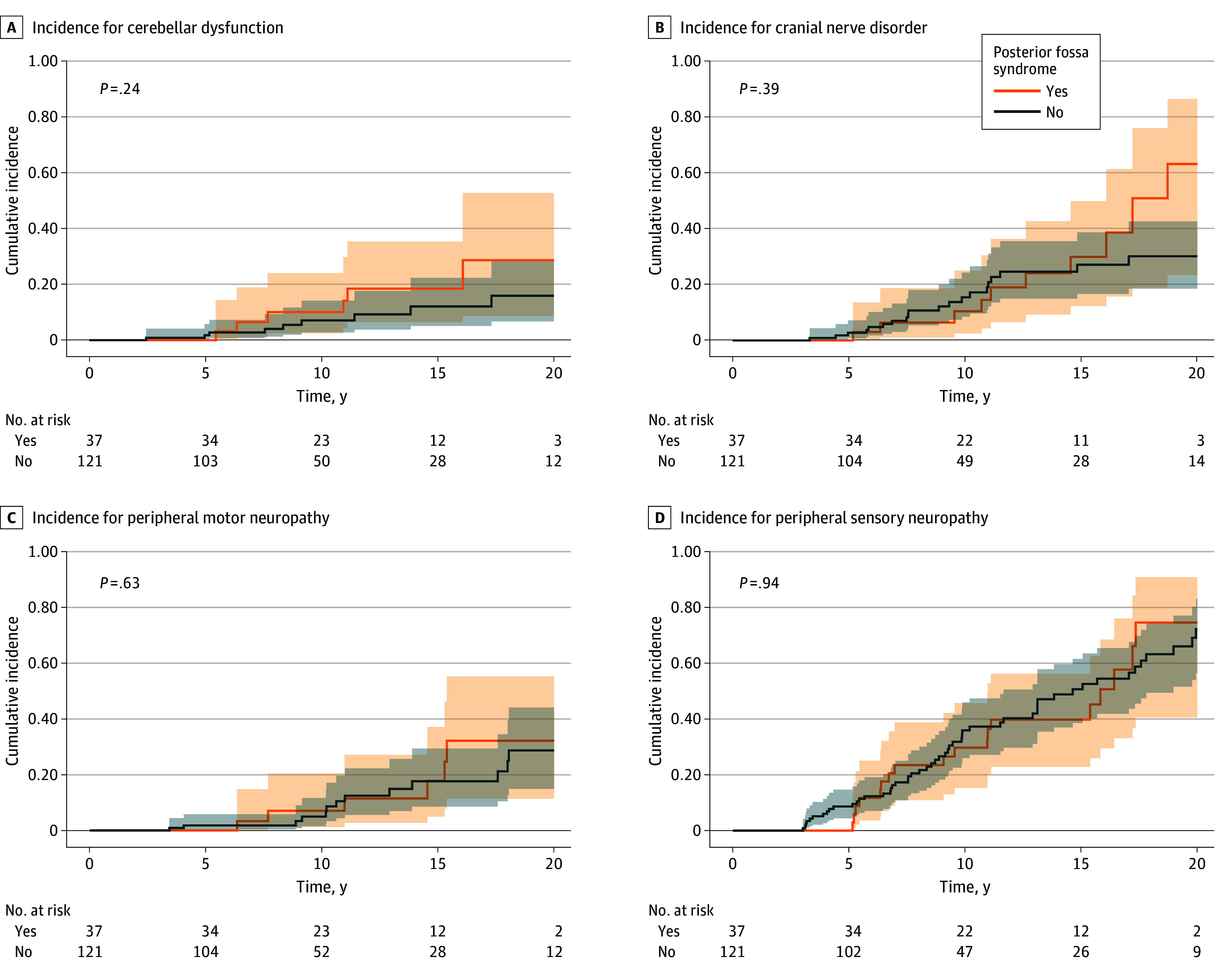
Cumulative Incidence Function Plots of Grade 2 or Higher Neurologic Impairments Among Survivors With and Without Posterior Fossa Syndrome

### Long-Term Neurocognitive Impairment

Participants with history of posterior fossa syndrome demonstrated lower performance across several neurocognitive domains compared with those without posterior fossa syndrome (Table 2; eTable 4 in Supplement 1), including for focused attention (mean [SD] score, −2.7 [1.45] vs −1.1 [1.70]; *P* < .001), motor processing speed (mean [SD] score, −3.3 [1.15] vs −2.2 [1.50]; *P* < .001), visuomotor processing speed (mean [SD] score, −2.3 [0.83] vs −1.3 [0.93]; *P* < .001), cognitive flexibility (mean [SD] score, −3.4 [1.20] vs −1.9 [1.77]; *P* < .001), verbal fluency (mean [SD] score, −1.4 [1.03] vs −0.7 [1.18]; *P* = .001), visual memory (mean [SD] score, −1.9 [1.33] vs −1.3 [1.30]; *P* = .02), and working memory (mean [SD] score, −1.1 [1.02] vs −0.6 [1.06]; *P* = .01). After adjusting for demographic and treatment variables, posterior fossa syndrome remained significantly associated with poorer focused attention (β = −1.04 [95% CI, −1.62 to −0.45]), motor processing speed (β = −0.62 [95% CI, −1.16 to −0.09]), visuomotor processing speed (β = −0.65 [95% CI, −0.97 to −0.33]), and cognitive flexibility (β = −0.85 [95% CI, −1.44 to −0.27]). Differences in verbal fluency and visual memory were not statistically significant. No adjusted differences were identified for general intelligence, academic achievement, or most Neurocognitive Questionnaire self-report domains. In multivariable models, posterior fossa syndrome, younger age at diagnosis, and CSF shunt placement were associated with lower scores in selected domains (eTables 5-8 in Supplement 1).

**Table 2.** Neurocognitive Outcomes Comparing Survivors of Medulloblastoma With and Without Posterior Fossa Syndrome, With Corresponding Adjusted Difference in *z* Score

Domain / Outcome	Mean (SD) score		Difference in *z* score (95% CI)^a^	*P* value
	Posterior fossa syndrome (n = 37)	No posterior fossa syndrome (n = 121)		
Attention				
Focused attention	−2.7 (1.45)	−1.1 (1.70)	−1.04 (−1.62 to −0.45)	<.001
Sustained attention	−1.2 (1.76)	−0.5 (1.44)	−0.45 (−1.07 to 0.16)	.15
Variability	−0.8 (1.50)	−0.4 (1.23)	−0.16 (−0.69 to 0.37)	.54
Processing speed				
Motor processing speed	−3.3 (1.15)	−2.2 (1.50)	−0.62 (−1.16 to −0.09)	.02
Visuomotor processing speed	−2.3 (0.83)	−1.3 (0.93)	−0.65 (−0.97 to −0.33)	<.001
Executive function				
Cognitive flexibility	−3.4 (1.20)	−1.9 (1.77)	−0.85 (−1.44 to −0.27)	.005
Verbal fluency	−1.4 (1.03)	−0.7 (1.18)	−0.42 (−0.86 to 0.02)	.06
Self-monitoring	−0.6 (1.16)	−0.3 (1.20)	−0.22 (−0.68 to 0.24)	.35
General intelligence				
Full-scale IQ	−1.2 (1.21)	−0.8 (1.07)	−0.06 (−0.45 to 0.33)	.75
Nonverbal reasoning	−1.1 (1.17)	−0.7 (1.08)	−0.11 (−0.52 to 0.29)	.57
Vocabulary and verbal reasoning	−1.0 (1.16)	−0.8 (1.07)	0.00 (−0.39 to 0.38)	.99
Academics				
Mathematics	−1.6 (1.37)	−1.3 (1.24)	0.07 (−0.36 to 0.50)	.75
Reading	−1.3 (1.24)	−0.8 (1.07)	−0.27 (−0.67 to 0.13)	.18
Memory				
Verbal learning	−1.3 (1.32)	−1.0 (1.28)	−0.16 (−0.66 to 0.35)	.54
Short-term memory	−0.9 (0.99)	−0.8 (1.08)	0.01 (−0.40 to 0.41)	.97
Long-term memory	−1.3 (1.39)	−0.9 (1.28)	−0.23 (−0.74 to 0.29)	.39
Visual memory	−1.9 (1.33)	−1.3 (1.30)	−0.44 (−0.96 to 0.07)	.09
Working memory	−1.1 (1.02)	−0.6 (1.06)	−0.25 (−0.66 to 0.15)	.21
NCQ self-report				
Memory	1.2 (1.04)	1.4 (1.39)	−0.33 (−0.88 to 0.23)	.24
Task efficiency	1.7 (1.09)	1.1 (1.31)	0.23 (−0.28 to 0.75)	.37
Emotional regulation	0.5 (1.01)	0.3 (1.13)	0.06 (−0.38 to 0.51)	.77
Organization	0.5 (0.98)	0.3 (1.03)	0.23 (−0.21 to 0.67)	.30

Abbreviation: NCQ, Neurocognitive Questionnaire.

^a^ Adjusted for age at diagnosis, years since diagnosis, sex, extent of resection, craniospinal radiation dose, cerebrospinal fluid shunt, treatment protocol, and hearing loss. The missing data were less than 14% for each independent outcome.

### Long-Term Physical Performance, Social Outcomes, and Quality of Life Outcomes

Participants with posterior fossa syndrome had poorer Physical Performance Test *z* scores (estimate, −3.65 [95% CI, −5.36 to −1.93]; *P* < .001) than those without posterior fossa syndrome after adjusting for age at diagnosis, years from diagnosis, sex, surgical resections, craniospinal radiation, shunt placement, and treatment protocol (Table 3). Participants with posterior fossa syndrome were more likely to require assistance with routine needs (eg, walking, grocery shopping, cleaning) (OR, 8.00 [95% CI, 2.56 to 25.04]; *P* < .001) (Table 4). Independent living was reported by 7 participants (22.6%) with posterior fossa syndrome compared with 36 participants (35.3%) without posterior fossa syndrome (Table 4). Although participants with posterior fossa syndrome reported higher unemployment (31 participants [64.7%]) than those without posterior fossa syndrome (64 participants [28.8%]) (*P* = .005) (Table 4), multivariable models adjusting for age at diagnosis, years from diagnosis, sex, surgical resections, craniospinal radiation dose, CSF shunt, and treatment protocol found no significant difference between groups (OR, 0.32 [95% CI, 0.08 to 1.27]; *P* = .11) (Table 4). Participants with posterior fossa syndrome were less likely to have a driver’s license (11 participants [39.3%]) than those without posterior fossa syndrome (72 participants [70.6%]) (*P* = .002), a difference which was not significant in multivariable models (OR, 0.42 [95% CI, 0.14 to 1.22]; *P* = .57) (Table 4).

**Table 3.** Multivariable Models Physical Performance Measures in Survivors of Medulloblastoma With and Without Posterior Fossa Syndrome

Variable	Mean (SD)		Adjusted mean difference, β (95% CI)^a^	*P* value
	Posterior fossa syndrome (n = 37)	No posterior fossa syndrome (n = 121)		
Physical Performance Test, total test score	20.1 (5.91)	24.7 (3.27)	−3.65 (−5.36 to −1.93)	<.001
Resting heart rate	78.2 (14.80)	78.3 (12.63)	−0.42 (−5.19 to 4.35)	.86
Mobility	8.7 (3.43)	7.9 (6.65)	0.43 (−2.11 to 2.97)	.73

^a^ Adjusted for age at diagnosis, years since diagnosis, sex, surgical resections, craniospinal radiation dose, cerebrospinal fluid shunt, and treatment protocol. The missing data were less than 15% for each independent outcome.

**Table 4.** Social, Functional, and Quality of Life Outcomes Among Survivors of Medulloblastoma With and Without Posterior Fossa Syndrome

Outcome	No. (%)		Adjusted, OR (95% CI)^a^	*P* value
	Posterior fossa syndrome (n = 37)	No posterior fossa syndrome (n = 121)		
Assistance with routine needs	17 (60.7)	18 (17.6)	8.00 (2.56 to 25.04)	<.001^b^
Assistance with personal care needs	3 (10.7)	3 (2.9)	3.06 (0.39 to 24.27)	.29^c^
Driver’s license	11 (39.3)	72 (70.6)	0.42 (0.14 to 1.22)	.57^b^
Educational attainment				
≥College	6 (22.2)	28 (28.3)	0.77 (0.31 to 1.89)	.57^b^
≥High school	8 (29.6)	35 (35.3)		
<High school	13 (48.1)	36 (36.4)		
Employed, yes	6 (35.3)	57 (71.2)	0.32 (0.08 to 1.27)	.11^b^
Independent living	7 (22.6)	36 (35.3)	0.66 (0.17 to 2.47)	.54^c^
Married, yes	5 (50.0)	20 (50.0)	0.50 (0.11 to 2.37)	.38^c^
HRQoL				
Physical Component Summary impairment^d^^,^^e^				
Overall	10 (37.0)	17 (17.2)	2.13 (0.71 to 6.44)	.18^b^
Physical functioning subscale^d^^,^^e^	11 (39.3)	20 (19.6)	1.61 (0.57 to 4.54)	.37^b^
Role limitations (physical)^d^^,^^e^	12 (42.9)	25 (24.5)	1.98 (0.71 to 5.52)	.19^b^
Bodily pain impairment^d^^,^^e^	3 (10.7)	11 (11.0)	0.70 (0.14 to 3.38)	.65^c^
General health impairment^d^^,^^e^	6 (20.7)	21 (20.8)	1.61 (0.50 to 5.25)	.43^b^
Mental Component Summary (MCS) impairment^d^^,^^e^				
Overall	4 (14.8)	12 (12.1)	1.12 (0.26 to 4.87)	.88^c^
Vitality impairment^d^^,^^e^	3 (10.3)	14 (13.9)	0.88 (0.19 to 4.09)	.87^c^
Social functioning impairment^d^^,^^e^	6 (20.7)	13 (12.7)	1.32 (0.39 to 4.49)	.67^c^
Role limitations (emotional)^d^^,^^e^	6 (21.4)	8 (7.8)	3.31 (0.79 to 13.84)	.10^c^
Emotional well-being impairment^d^^,^^e^	4 (13.8)	12 (12.0)	1.00 (0.25 to 3.93)	.99^b^
Alcohol use disorder risk^d^^,^^e^^,^^f^	5 (16.7)	26 (25.7)	NA	.30^b^
Smoking status: current smoker	5 (16.7)	7 (6.8)	NA	.14^b^
Physical activity^g^	13 (43.3)	41 (41.0)	NA	.82^b^

Abbreviations: HRQoL, health-related quality of life; OR, odds ratio.

^a^ ORs represent the association between posterior fossa syndrome (vs non-posterior fossa syndrome) and each outcome. Multivariable models adjusted for age at diagnosis, years since diagnosis, sex, number of surgical resections, craniospinal radiation dose, cerebrospinal fluid shunt, and treatment protocol.

^b^ *P* values calculated using the χ^2^ test.

^c^ *P* values calculated using Fisher exact test.

^d^ Impairment on Short Form-36 domains was defined as a score less than 40 (1 SD below the normative mean).

^e^ Missing values were excluded from analyses. The missing data are less than 20% for each independent outcome.

^f^ Self-reported alcohol use. Alcohol use disorder is defined per National Institute on Alcohol Abuse and Alcoholism criteria (>3 drinks/day or >7 drinks/week for females or >4 drinks/day or >14 drinks/week for males).

^g^ Physical activity based on the National Health and Nutrition Examination Survey Physical Activity Questionnaire; meeting Centers for Disease Control and Prevention criteria was defined as at least ≥150 minutes/week of moderate or vigorous activity.

## Discussion

Our cohort study identified significant neurocognitive and physical impairments in participants with posterior fossa syndrome at a median of 14.2 years of follow-up, which may adversely affect their social and functional abilities.^49^ After adjusting for age at diagnosis and treatment, participants with posterior fossa syndrome demonstrated greater neurocognitive impairments in attention, processing speed, and executive function than those without posterior fossa syndrome. Additionally, participants with posterior fossa syndrome demonstrated decreased physical performance scores and increased need for assistance with daily routine needs, highlighting the long-term outcomes of the devastating postoperative complication of posterior fossa syndrome. Our findings complement but differ from those of Schreiber et al,^27^ who described worsening neurocognitive outcomes within approximately 5 years of diagnosis among individuals treated with the SJMB03 risk-adapted craniospinal irradiation protocol. In contrast, our study draws on the broader SJLIFE cohort, which includes survivors of medulloblastoma diagnosed from 1985 to 2012 across multiple treatment protocols and regimens. We found that neurocognitive impairments not only persisted but broadened over time, with enduring deficits in attention, processing speed, and executive function evident well into adulthood. These results extend prior observations of early survivorship by suggesting that neurocognitive dysfunction in individuals with posterior fossa syndrome is long-lasting, clinically meaningful, and more extensive than previously characterized.

Impaired processing speed (>2 SD below the mean) and below-average intellectual ability have been observed in individuals with posterior fossa syndrome as early as 1 year after diagnosis,^50^ with deficits persisting into later survivorship, likely reflecting disruption of frontocerebellar pathways.^11^ Our findings suggest that more than a decade after diagnosis, individuals with posterior fossa syndrome continue to have poorer attention, processing speed, and executive functioning than survivors of medulloblastoma without posterior fossa syndrome. These findings suggest that early brain injury limits brain plasticity and cognitive recovery in the long term. Additionally, we found discrepancies between objective and subjective neurocognitive outcomes, indicating that survivors may underreport their impairments, possibly due to limited insight or impaired judgment, as reported in adult brain tumor patients.^51,52,53^

Participants with posterior fossa syndrome had a higher shunt rate than those without posterior fossa syndrome, consistent with increased posterior fossa syndrome risk.^4^ Hydrocephalus is associated with reduced attention, IQ, academic performance, and school retention in childhood brain tumor survivors.^54^ Although the incidence of hydrocephalus appears similar across surgical centers, shunt placement is reported more frequently at lower-volume centers.^4,55,56^ Higher neurosurgical volume has been associated with improved outcomes. However, definitions vary across studies and over time,^55,57,58^ with Nationwide Inpatient Sample data indicating a progressive centralization of pediatric brain tumor resections to higher-volume centers beginning in the late 1980s. In 2021, Khan et al^4^ defined low-volume centers as those in resource-limited settings, staffed by nonpediatric neurosurgeons, or with cumulative pediatric neurosurgical case volume of fewer than 500 procedures, whereas accredited pediatric neurosurgery fellowships were considered high-volume regardless of caseload.^59^ Given these differing frameworks and the extended temporal span of our cohort, volume-based comparisons should be interpreted cautiously. Even after controlling for shunt placement, posterior fossa syndrome remained associated with poorer cognitive outcomes.

Although not statistically significant, numerically more participants with posterior fossa syndrome patients required multiple surgical resections (15 participants [40.5%] vs 30 participants [24.8%]; *P* = .06). Long-term complications can be reduced by performing surgical procedures at high-volume centers staffed by accredited pediatric neurosurgeons.^60^ One high-volume center observed reduced incidence of posterior fossa syndrome, from 39% to 10.8%, in association with using a telovelar-over-transvermian approach.^3^ However, a large prospective European postoperative speech impairment study found no association between surgical approach and postoperative speech impairment.^8^ Recent consensus guidelines recommend approaches that maximize exposure while minimizing retraction, even if a small residual is left to preserve function.^61^

Hearing loss and neurological impairments are known treatment-related comorbidities in patients treated for medulloblastoma secondary to exposure to platinum-based chemotherapies and radiation. Based on our findings, we cannot determine whether posterior fossa syndrome was associated with increasing these risks. Severe hearing loss, younger age at diagnosis, and posterior fossa syndrome are substantial risk factors associated with decline in intellectual and academic outcomes in childhood medulloblastoma.^62,63^ This emphasizes the need for close monitoring and early interventions, such as hearing aids and reading interventions, in patients with posterior fossa syndrome at risk of neurocognitive impairments. However, prior studies indicate that patients with posterior fossa syndrome experience more severe neurological deficits and slower recovery in the first 5 years after diagnosis compared with those without posterior fossa syndrome.^4^

Notably, prior studies of pediatric survivors of brain tumor found that only 40% achieved complete independence,^64^ and many faced work-related challenges.^65^ In contrast, only 22.6% of participants with posterior fossa syndrome in our study lived independently, potentially underscoring the additive burden of posterior fossa syndrome alongside craniospinal radiation. However, we used a single indicator of independent living, whereas prior studies used multiple indicators in their definitions of independence. Individuals with posterior fossa syndrome are nearly twice as likely to require special education services compared with those without posterior fossa syndrome.^66^ Our findings highlight significant deficits in quality of life, social outcomes, and functioning. Early cognitive assessments can help identify long-term needs, guiding caregiver education and support services.^67^ This underscores the importance of educational institutions and employers recognizing these unique challenges and providing necessary accommodations for individuals with posterior fossa syndrome.^68^

Evidence for the efficacy of cognitive remediation in posterior fossa syndrome is still emerging, but initial findings show promise. A pilot study demonstrated the benefits of a cognitive-motor intervention, resulting in significant improvements in visual attention, visuospatial working memory, semantic verbal fluency, eye-hand coordination, reaction time, and auditory-motor synchronization and a reduction in ataxia symptoms.^69^ Interventions focused on physical activity and health behaviors also benefit brain structure and functioning among survivors.^70,71^

### Limitations

This study has notable strengths, but several limitations highlight areas that warrant further research. Posterior fossa syndrome diagnoses were retrospectively made from medical records of patients diagnosed between 1962 and 2012, before the syndrome was well characterized and severity classifications existed, potentially underestimating milder cases. The first study linking mutism to cerebellar injury was published in 1985,^14^ and a formal definition of posterior fossa syndrome was only established in 2011.^72^ The current posterior fossa syndrome diagnostic criteria^4,73,74,75^ categorize impairments into complete or partial mutism, providing a more granular measure of posterior fossa syndrome.^4^ We were unable to include molecular subgroup data of medulloblastoma because our patient diagnoses predated the 2012 consensus.^76^ Records lacked details on tumor size and surgical techniques, preventing inclusion in our analysis. As standardized motor coordination measures, such as the Sensory Organization Test and modified Total Neuropathy Score, were not collected, our ability to characterize gait and coordination deficits was limited—a gap that future studies can address using our dedicated gait laboratory. Furthermore, small sample size and reliance on the most recent assessment restricted our analysis to cross-sectional long-term outcomes, which may not capture progression or account for selection bias.

## Conclusions

In this cohort study, survivors of medulloblastoma with posterior fossa syndrome experienced persistent neurocognitive impairments and diminished physical performance, which were associated with greater need for assistance with daily activities. While acute postsurgical deficits associated with posterior fossa syndrome are recognized, their persistence in long-term survivors highlights the importance of longitudinal surveillance and continued intervention to optimize quality of life. These findings suggest that posterior fossa syndrome could worsen neurocognitive and neurological outcomes in survivors of medulloblastoma. Early assessment of posterior fossa syndrome severity in this high-risk population can facilitate timely opportunities for caregiver education, speech and physical therapy, and advocacy for support services, including academic support, vocational rehabilitation, and accommodations that enhance functional independence and quality of life. Finally, our study highlighted the substantial and long-lasting morbidity associated with a postoperative complication, emphasizing the urgent need for improved surgical techniques in posterior fossa tumor resections performed at high-volume, accredited, pediatric neurosurgical centers.

## Notes

Supplement 1. eMethods. eFigure. Flow diagram of study cohort selection eTable 1. Comparison between participants and non-participants eTable 2. Neurologic impairments (CTCAE grade ≥2) among survivors with and without PFS eTable 3. Multivariable Models testing differences in neurologic impairment between PFS and non-PFS survivors eTable 4. Neurocognitive tests used to study outcomes eTable 5. Multivariable models testing differences in visuo-motor processing speed between PFS and non-PFS survivors eTable 6. Multivariable models testing differences in focused attention between PFS and non-PFS survivors eTable 7. Multivariable models testing differences in motor processing between PFS and non-PFS survivors eTable 8. Multivariable models testing differences in cognitive flexibility z-scores between PFS and non-PFS survivors

Supplement 2. Data Sharing Statement
