# Supplementary material for: Long-Term Outcomes Associated With Posterior Fossa Syndrome in Survivors of Childhood Medulloblastoma
Source: JAMA Netw Open. 2026 Feb 19;9(2):e2559376. doi: 10.1001/jamanetworkopen.2025.59376 (PMC12921523; doi:10.1001/jamanetworkopen.2025.59376)
Supplement: Supplement 1. — eMethods. eFigure. Flow diagram of study cohort selection eTable 1. Comparison between participants and non-participants eTable 2. Neurologic impairments (CTCAE grade ≥2) among survivors with and without PFS eTable 3. Multivariable Models testing differences in neurologic impairment between PFS and non-PFS survivors eTable 4. Neurocognitive tests used to study outcomes eTable 5. Multivariable models testing differences in visuo-motor processing speed between PFS and non-PFS survivors eTable 6. Multivariable models testing differences in focused attention between PFS and non-PFS survivors eTable 7. Multivariable models testing differences in motor processing between PFS and non-PFS survivors eTable 8. Multivariable models testing differences in cognitive flexibility z-scores between PFS and non-PFS survivors [file jamanetwopen-e2559376-s001.pdf]

Supplemental Online Content

Sarvode S, Dhaduk R, Chen Y, et al. Long-term outcomes associated with posterior fossa syndrome in survivors of childhood medulloblastoma. *JAMA Netw Open*. 2026;9(2):e2559376. doi:10.1001/jamanetworkopen.2025.59376

eMethods.

eFigure. Flow diagram of study cohort selection

eTable 1. Comparison between participants and non-participants

eTable 2. Neurologic impairments (CTCAE grade ≥2) among survivors with and without PFS

eTable 3. Multivariable Models Testing Differences in Neurologic Impairment Between PFS and non-PFS survivors

eTable 4. Neurocognitive tests used to study outcomes

eTable 5. Multivariable Models Testing Differences in Visuo-Motor Processing Speed Between PFS and Non-PFS Survivors

eTable 6. Multivariable Models Testing Differences in Focused Attention Between PFS and Non-PFS Survivors

eTable 7. Multivariable Models Testing Differences in Motor Processing Between PFS and Non-PFS Survivors

eTable 8. Multivariable Models Testing Differences in Cognitive Flexibility Z-Scores Between PFS and Non-PFS Survivors

This supplemental material has been provided by the authors to give readers additional information about their work.

© 2026 Sarvode S et al. *JAMA Network Open*.

eMethods. Physical Performance Methods from the Human Performance Laboratory:

The physical performance methods used in this study are adapted from those previously described.[43] The 6-minute walk test (6MWT) assesses aerobic capacity. Before the test, participants were asked to sit quietly for five minutes to acquire a resting heart rate. Participants were then instructed to walk as fast as they could around a 41-meter track. Heart rate (HR), oxygen saturation, and rating of perceived exertion were measured using the Masimo Rad-5 handheld pulse oximeter (Irvine, CA), and then re-tested at 2, 4, and 6 minutes and at 2 minutes into recovery. The 6MWT has been validated to assess cardiorespiratory fitness in childhood cancer survivors. The total distance in meters was documented.

Lower back and hamstring flexibility was assessed using a Flex-Tester sit-and-reach box (Novel Products, Inc., Rockton, IL).[43] Participants sat with their hips at 90°, legs extended, and knees locked. Participants were asked to flex only at the hips and reach forward as far as possible. The test was repeated, and the best distance to the nearest 0.5 cm was recorded. Isokinetic knee extension strength and endurance were measured in a seated position using a Biodex III dynamometer (Biodex Medical Systems, Shirley, NY). Strength was measured as peak torque (N·m/kg) from five repetitions[40] at 60° per second, and endurance was expressed as the difference between average peak torque from the first three and the last three of 15 repetitions at 300° per second. Dorsiflexion and plantarflexion peak torque were measured as peak torque (N·m/kg) from five repetitions at 60° per second.[43]

© 2026 Sarvode S et al. *JAMA Network Open*.

eFigure 1. Flow diagram of study cohort selection.

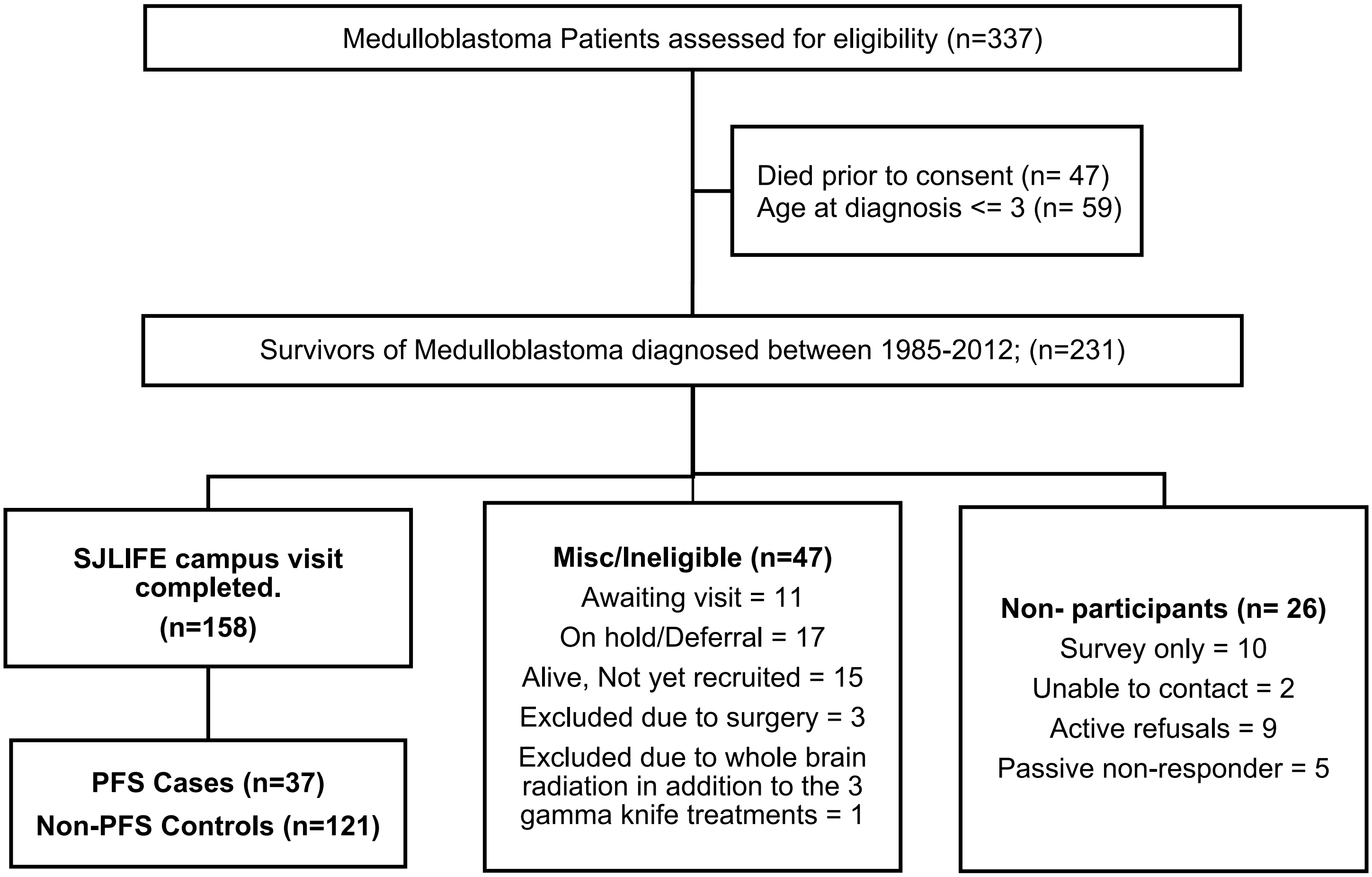

The diagram shows the number of medulloblastoma survivors screened, excluded, and included in the final analysis, along with the reasons for exclusion at each step.

Abbreviations: SJLIFE, St. Jude Lifetime Cohort Study, PFS, Posterior Fossa Syndrome

© 2026 Sarvode S et al. *JAMA Network Open*.

eTable 1: Comparison between participants and non-participants

	Participants (N=158)	Non- Participants(N=73)	p-value
**Sex**, n (%)			.35[a]
Female	62 (39.2%)	24 (32.9%)	
Male	96 (60.8%)	49 (67.1%)	
**Age at diagnosis**, Median (Range)	8.8 (3.0-22.3)	7.5 (3.1-21.6)	.18[b]
**Race**, n (%)*			.07[a]
Other	43 (27.2%)	12 (16.4%)	
White non-Hispanic	115 (72.8%)	61 (83.6%)	
**Craniospinal Radiation**, n (%)			.24[a]
< 30 Gy	84 (53.2%)	36 (62.1%)	
≥ 30 Gy	74 (46.8%)	22 (37.9%)	
**No. of Surgical Resections**, n (%)			.81[a]
1	113 (71.5%)	41 (73.2%)	
> 1	45 (28.5%)	15 (26.8%)	
**Shunt**, n (%)	58 (36.7%)	31 (42.5%)	.42[a]

a X^2 test p-value.

b Mann–Whitney p-value:

Abbreviations: BMI, body mass index; Gy, gray; CSI, craniospinal irradiation; PFS, posterior fossa syndrome.

© 2026 Sarvode S et al. *JAMA Network Open*.

eTable 2: Neurologic impairments (CTCAE grade ≥2) among survivors with and without PFS.

Neurologic impairment	PFS (N=37)	Non-PFS (N=121)	p-value
Cerebellar dysfunction, n (%)	7 (18.9%)	10 (8.3%)	.08[a]
Cranial nerve disorder, n (%)	10 (27.0%)	20 (16.5%)	.15[b]
Dysarthria, n (%)	1 (2.7%)	0 (0.0%)	.23[a]
Headaches, n (%)	1 (2.7%)	12 (9.9%)	.30[a]
Movement disorders, n (%)	0 (0.0%)	1 (0.8%)	1.00[a]
Paralytic disorder, n (%)	1 (2.7%)	5 (4.1%)	1.00[a]
Peripheral motor neuropathy, n (%)	7 (18.9%)	15 (12.4%)	.32[a]
Peripheral sensory neuropathy, n (%)	20 (54.0%)	55 (45.5%)	.36[b]
Seizure, n (%)	3 (8.1%)	11 (9.1%)	1.00[a]
Hearing loss (SIOP grade 3 and above)	32 (86.5%)	80 (66.1%)	.02[b]

a Fisher's exact p-value

b X^2 test p-value

Abbreviations: CTCAE, Common Terminology Criteria for Adverse Events; PFS, posterior fossa syndrome; SIOP, International Society of Pediatric Oncology.

© 2026 Sarvode S et al. *JAMA Network Open*.

eTable 3: Multivariable Models Testing Differences in Neurologic Impairment Between PFS and non-PFS survivors

Variable	Odds Ratio (95% CI)	P value
Cranial nerve disorder	1.63 (0.67-4.01)	.28
Cerebellar dysfunction	1.97 (0.68-5.74)	.21
Dysarthria	6.67 (0.55-80.49)	.13
Headaches	0.50 (0.08-2.99)	.45
Movement disorders	0.65 (0.05-7.95)	.73
Paralytic disorder	0.54 (0.09-3.11)	.49
Peripheral motor neuropathy	1.62 (0.49-5.34)	.42
Peripheral sensory neuropathy	1.57 (0.69-3.56)	.28
Seizure	0.97 (0.32-2.98)	.96
Hearing loss (SIOP grade 3 and above)	1.84 (0.58-5.82)	.30
Shunt	1.89 (0.14-25.85)	.63

Other variables adjusted for in the model are Age at diagnosis, Years since diagnosis, Sex, Surgical resections, Cerebrospinal radiation, Surgical shunt, and Treatment protocol.

Abbreviations: CI, confidence interval; OR, odds ratio; CTCAE, Common Terminology Criteria for Adverse Events; PFS, posterior fossa syndrome; SIOP, International Society of Pediatric Oncology

© 2026 Sarvode S et al. *JAMA Network Open*.

eTable 4: Neurocognitive tests used to study outcomes.

**Domain**	**Neurocognitive test**
**Attention**	
Focused Attention	Trail Making Test Part A
Sustained Attention	Conners' CPT 2/CPT 3 Omissions
Variability	Conners' CPT 2/CPT 3 Variability
**Processing Speed**	
Motor Processing Speed	Grooved Pegboard Dominant Hand
Visuo-Motor Processing Speed	Wechsler Intelligence Scale Digit Symbol-Coding
**Executive Function**	
Cognitive Flexibility	Trail Making Test Part B
Verbal Fluency	Controlled Oral Word Association Test
Self-Monitoring	Conners' CPT 2/CPT 3 Commissions
**General Intelligence**	
Full Scale IQ	WASI/WASI-2 Full Scale IQ
Non-Verbal Reasoning	WASI/WASI-2 Matrix Reasoning
Vocabulary	WASI/WASI-2 Vocabulary
**Academics**	
Mathematics	W-J-III NU Calculation
Reading	W-J-III NU Letter-Word Identification
**Memory**	
Verbal Learning	CVLT Trials 1-5 Total
Short-Term Memory	Wechsler Intelligence Scale Longest Digit Span Forward Total
Long-Term Memory	CVLT Long Delay Free Recall
Visual Memory	TOMAL-2 Visual Selective Reminding
Working Memory	Wechsler Intelligence Scale Digit Span Backward Total
**NCQ Self-report**[34]	

© 2026 Sarvode S et al. *JAMA Network Open*.

Memory	NCQ 25-item (Memory
Task Efficiency	NCQ 25-item Task Efficiency
Emotional Regulation	NCQ 25-item Emotional Regulation
Organization	NCQ 25-item Organization

All measures are standard neurocognitive batteries that use non-cancer population norms.

Abbreviations: CPT, Continuous Performance Test; WAIS, Wechsler Adult Intelligence Scale; WISC, Wechsler Intelligence Scale for Children; CVLT, California Verbal Learning Test; WJ, Woodcock–Johnson Tests; TOMAL, Test of Memory and Learning; NCQ, Neurocognitive Questionnaire.

© 2026 Sarvode S et al. *JAMA Network Open*.

eTable 5: Multivariable Models Testing Differences in Visuo-Motor Processing Speed Between PFS and Non-PFS Survivors

**Variable**	**Difference in Z-score (95% CI)**	**P value**
PFS (Yes vs No)	-0.63 (-0.95 to -0.31)	<.001
Time since diagnosis (Years)	-0.02 (-0.05 to 0.02)	.34
Age at diagnosis	0.06 (0.03 to 0.09)	<.001
Sex (Female vs Male)	-0.09 (-0.35 to 0.18)	.51
Treatment protocol (Other vs SJMB96)	0.09 (-0.31 to 0.50)	.65
Treatment protocol (SJMB03 vs SJMB96)	0.21 (-0.19 to 0.61)	.30
Surgical resections (number of resections)	-0.04 (-0.33 to 0.26)	.79
Craniospinal radiation (< 30 Gy vs ≥ 30 Gy)	0.17 (-0.12 to 0.47)	.24
CSF shunt (Yes vs No)	-0.59 (-0.87 to -0.31)	<.001
Hearing loss (SIOP grade 3 and above)	-0.12(-0.44 to 0.20)	.45

Abbreviations: CI, confidence interval; PFS, posterior fossa syndrome; SJMB-96 - St Jude Medulloblastoma-96; SJMB- 03 - St Jude Medulloblastoma-03; Gy- gray; CSF- Cerebro Spinal Fluid; SIOP, International Society of Pediatric Oncology

© 2026 Sarvode S et al. *JAMA Network Open*.

eTable 6: Multivariable Models Testing Differences in Focused Attention Between PFS and Non-PFS Survivors

Variable	Difference in Z-score (95% CI)	P value
PFS (Yes vs No)	-1.05 (-1.64 to -0.46)	<.001
Time since diagnosis (Years)	-0.03 (-0.10 to 0.03)	.35
Age at diagnosis	0.14 (0.08 to 0.19)	<.001
Sex (Female vs Male)	-0.46 (-0.95 to 0.03)	.06
Treatment protocol (Other vs SJMB96)	0.16 (-0.59 to 0.91)	.67
Treatment protocol (SJMB03 vs SJMB96)	0.08 (-0.66 to 0.82)	.83
Surgical resections (number of resections)	-0.32 (-0.86 to 0.23)	.25
Craniospinal radiation (< 30 Gy vs ≥ 30 Gy)	0.30 (-0.24 to 0.84)	.27
CSF shunt (Yes vs No)	-0.81 (-1.33 to -0.28)	.003
Hearing loss (SIOP grade 3 and above)	0.08 (-0.51 to 0.68)	.78

Abbreviations: CI, confidence interval; PFS, posterior fossa syndrome; SJMB-96 - St Jude Medulloblastoma-96; SJMB- 03 - St Jude Medulloblastoma-03; Gy- gray; CSF- Cerebro Spinal Fluid; SIOP, International Society of Pediatric Oncology

© 2026 Sarvode S et al. *JAMA Network Open*.

eTable 7: Multivariable Models Testing Differences in Motor Processing Between PFS and Non-PFS Survivors

Variable	Difference in Z-score (95% CI)	P value
PFS (Yes vs No)	-0.60 (-1.13 to -0.06)	.03
Time since diagnosis (Years)	-0.01 (-0.07 to 0.05)	.75
Age at diagnosis	0.01 (-0.04 to 0.06)	.63
Sex (Female vs Male)	0.03 (-0.41 to 0.48)	.89
Treatment protocol (Other vs SJMB96)	-0.01 (0.69 to 0.68)	.98
Treatment protocol (SJMB03 vs SJMB96)	0.66 (0.01 to 1.32)	.05
Surgical resections (number of resections)	0.06 (-0.44 to 0.55)	.83
Craniospinal radiation (< 30 Gy vs ≥ 30 Gy)	-0.05 (-0.54 to 0.44)	.84
CSF shunt (Yes vs No)	-0.96 (-1.43 to -0.49)	<.001
Hearing loss (SIOP grade 3 and above)	-0.24 (0.78 to 0.30)	.38

Abbreviations: CI, confidence interval; PFS, posterior fossa syndrome; SJMB-96 - St Jude Medulloblastoma-96; SJMB- 03 - St Jude Medulloblastoma-03; Gy- gray; CSF- Cerebro Spinal Fluid; SIOP, International Society of Pediatric Oncology

© 2026 Sarvode S et al. *JAMA Network Open*.

eTable 8: Multivariable Models Testing Differences in Cognitive Flexibility Z-Scores Between PFS and Non-PFS Survivors

Variable	Difference in Z-score (95% CI)	P value
PFS (Yes vs No)	-0.86 (-1.45 to -0.27)	.005
Time since diagnosis (Years)	-0.03 (-0.10 to 0.04)	.38
Age at diagnosis	0.10 (0.05 to 0.16)	<.001
Sex (Female vs Male)	-0.21 (-0.70 to 0.28)	.40
Treatment protocol (Other vs SJMB96)	0.47 (-0.28 to 1.21)	.22
Treatment protocol (SJMB03 vs SJMB96)	0.76 (0.02 to 1.50)	.04
Surgical resections (number of resections)	0.01 (-0.55 to 0.54)	.98
Craniospinal radiation (< 30 Gy vs ≥ 30 Gy)	-0.05 (-0.59 to 0.49)	.85
CSF shunt (Yes vs No)	-1.15 (-1.67 to -0.63)	<.001
Hearing loss (SIOP grade 3 and above)	0.04 (-0.55 to 0.64)	.89

Abbreviations: CI, confidence interval; PFS, posterior fossa syndrome; SJMB-96 - St Jude Medulloblastoma-96; SJMB- 03 - St Jude Medulloblastoma-03; Gy- gray; CSF- Cerebro Spinal Fluid; SIOP, International Society of Pediatric Oncology

© 2026 Sarvode S et al. *JAMA Network Open*.
