# Supplementary material for: Long-Term Outcomes Associated With Posterior Fossa Syndrome in Survivors of Childhood Medulloblastoma
Source: JAMA Netw Open. 2026 Feb 19;9(2):e2559376. doi: 10.1001/jamanetworkopen.2025.59376 (PMC12921523; doi:10.1001/jamanetworkopen.2025.59376)
Supplement: Supplement 2. — Data Sharing Statement [file jamanetwopen-e2559376-s002.pdf]

Data Sharing Statement

Sarvode. Long-Term Outcomes Associated With Posterior Fossa Syndrome in Survivors of Childhood Medulloblastoma. *JAMA Netw Open*. Published February 19, 2026.
doi:10.1001/jamanetworkopen.2025.59376

Data
Data available: Yes
Data types: Deidentified participant data
How to access data: The data supporting the findings of this study are available at: https://www.stjude.cloud/research-domains/cancer-survivorship. Data specific to this paper will be uploaded to https://zenodo.org upon manuscript publication.
When available: With publication

Supporting Documents
Document types: None

Additional Information
Who can access the data: anyone requesting the data
Types of analyses: for any purpose or for a specified purpose
Mechanisms of data availability: with a signed data access agreement
